# Genome wide copy number analyses of superficial esophageal squamous cell carcinoma with and without metastasis

**DOI:** 10.18632/oncotarget.13847

**Published:** 2016-12-10

**Authors:** Pengjiao Wang, Ling Shan, Liyan Xue, Bo Zheng, Jianming Ying, Ning Lu

**Affiliations:** ^1^ Department of Pathology, National Cancer Center/Cancer Hospital, Chinese Academy of Medical Sciences and Peking Union Medical College, Beijing, P. R. China

**Keywords:** superficial esophageal squamous cell carcinoma, copy number alterations, metastasis, microarray

## Abstract

Superficial esophageal squamous cell carcinoma (ESCC) is generally considered a subtype of less invasive ESCC. Yet a subset of these superficial ESCC would have metastasis after esophagostomy or endoscopic resection and lead to poor prognosis. The objective of this study is to determine biomarkers that can identify such subset of superficial ESCC that would have metastasis after surgery using genome wide copy number alteration (CNA) analyses. The CNAs of 38 cases of superficial ESCCs originated from radical surgery, including 19 without metastasis and 19 with metastasis within 5 years’ post-surgery, were analyzed using Affymetrix OncoScan™ FFPE Assay. A 39-gene signature was identified which characterized the subset of superficial ESCC with high risk of metastasis after surgery. In addition, recurrent CNAs of superficial ESCC were also investigated in the study. Amplification of 11q13.3 (*FGF4*) and deletion of 9p21.3 (*CDKN2A*) were found to be recurrent in all 38 superficial ESCCs analyzed. Notably amplifications of 3p26.33 (*SOX2OT*), 8q24.21 (*MYC*), 14q21.1 (*FOXA1*) and deletion of 3p12.1 (*GBE1*) were only found to be recurrent in metastaic superficial ESCCs. In conclusion, using CNAs analyses, we identify a 39-gene signature which characterizes the high risk metastatic superficial ESCCs and discover several recurrent CNAs that might be the driver alterations in metastasis among superficial ESCCs.

## INTRODUCTION

Esophageal squamous cell carcinoma (ESCC) is one of the most deadly tumors worldwide, with 5 year survival of only 10%. ESCC related death rates are particularly high in China, in part due to the lack of early screening tools and limited treatment options [1].

Superficial ESCC has significant better prognosis than the advanced ESCC, and can be treated effectively by endoscopic resection, including both mucosal resection and submucosal dissection [2, 3]. Endoscopic resection remains the most widely used treatment option for superficial ESCCs because it is safe and esophagus-preserving. However, 26-53% of superficial ESCCs have lymph node metastasis, and require additional esophagectomy or radiochemotherapy after endoscopic resection [4, 5]. Thus identifying biomarkers that can assess the metastatic risk in superficial ESCC is of particular importance.

Several clinicopathological features have been associated with high metastatic risk in superficial ESCC, including invasion depth, tumor size, angiolymphatic invasion and histological differentiation [4–6]. ESCCs are also characterized by CNAs [7, 8]. Among those characteristic CNAs, amplifications of 11q13.3-13.4 (*CCND1*), 3q26.33 (*SOX2*), 8q24.2 (*MYC*) and deletion of 3p14.2 (*FHIT*) have been associated with metastatic diseases [8–12]. Nevertheless, a comprehensive set of CNAs that can be used to predict metastasis risk in clinical samples are not yet available and would be important to select the appropriate treatment option for superficial ESCCs.

Genomic instabilities, in the form of chromosome instability (CIN), microsatellite instability (MIN) and point mutations, are characteristic for human cancers. CIN, including chromosome structural and number changes, is a major form of genomic instability [13]. According to their size, CNAs can be classified into focal or arm-level CNAs. Focal CNAs are very informative and often involve important oncogenes or tumor suppressor genes [14–17]. CNAs could be detected by next-generation sequencing, comparative array genomic hybridization or single nucleotide polymorphism (SNP) microarray. The OncoScan® assay is a SNP microarray including over 220,000 SNPs across human genome with increased probe density within 891 cancer related genes and are uniquely suited to detect subtle CNAs with high sensitivity and specificity.

Here we reported the analyses of 38 superficial ESCCs originated from radical surgery with both metastatic and metastasis-free samples with Affymetrix OncoScan™ array. The CNAs landscape of superficial ESCC were determined, and the focal recurrent CNAs were compared between the metastasis and metastasis-free cases.

## RESULTS

### Genome wide CNA of superficial ESCCs

**Figure 1:**
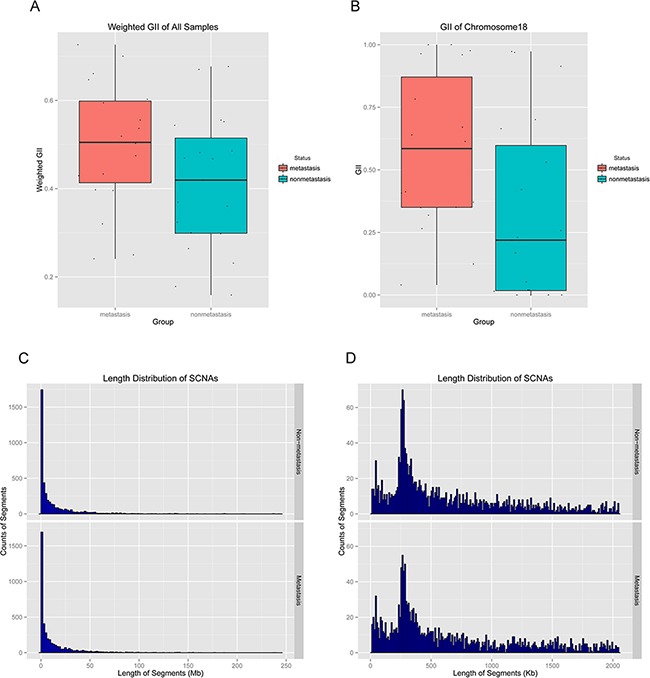
The whole genome copy number alteration (CNA) profiles of all 38 samples were shown **A**. Comparison of weighted genome instability index (GII) of all chromosomes between metastasis and non-metastasis groups (*p* = 0.07). **B**. Comparison of GII of Chromosome 18 between metastasis and non-metastasis groups (*p* = 0.03). **C**. Length distribution of all the CNAs in metastasis and non-metastasis groups. **D**. Length distribution of CNAs (< 2 Mb) in metastasis and non-metastasis groups.

The whole genome CNA profiles of all 38 samples were shown in Supplementary Figure S1. The microarray data has been deposited in the Gene Expression Omnibus (GEO) with the accession number GSE78926. Weighted GII was plotted to show the genome wide CNAs. Weighted GII was 45.5% in all superficial ESCC samples, and was 49.9% and 41.1% in metastasis group and non-metastasis group, respectively. The comparison of weighted GII in metastasis and non-metastasis group was shown in Figure 1A (*p* = 0.07). In addition, the comparison of GII on each chromosome in non-metastasis and metastasis groups was also performed. In non-metastasis group, chromosomes 3 (65.7%), 8 (78.2%) and 20 (59.8%) have the highest GII, whereas chromosomes 4 (29.5%), 12 (28.7%) and 21(25.2%) have lowest GII. In metastasis group, chromosomes 3 (75.2%), 8 (72.1%) and 14 (60.0%) have the highest GII, whereas chromosomes 15 (35.9%), 21 (31.8%) and 22 (38.1%) have the lowest GII. Chromosome 18 has higher GII in metastasis group than in the non-metastasis group (*p* = 0.03) (Figure 1B).

The average number of genome segments with CNAs in all 38 samples was 218, and the average length was 13.26 Mb (minimal length was 13.14 Kb and maximal length was 242.64 Mb). In metastasis group, the average number of segments with CNAs was 217, and the average length was 13.35 Mb (minimal length was 13.16 Kb and maximal length was 239.80 Mb). In non-metastasis group, the average number of segments with CNAs was 210, and the average length was 13.05 Mb (minimal length was 13.14 Kb and maximal length was 242.64 Mb). The distribution of size of CNAs length was shown in Figure 1C and 1D.

### Recurrent CNAs in metastasis and non-metastasis groups

**Figure 2:**
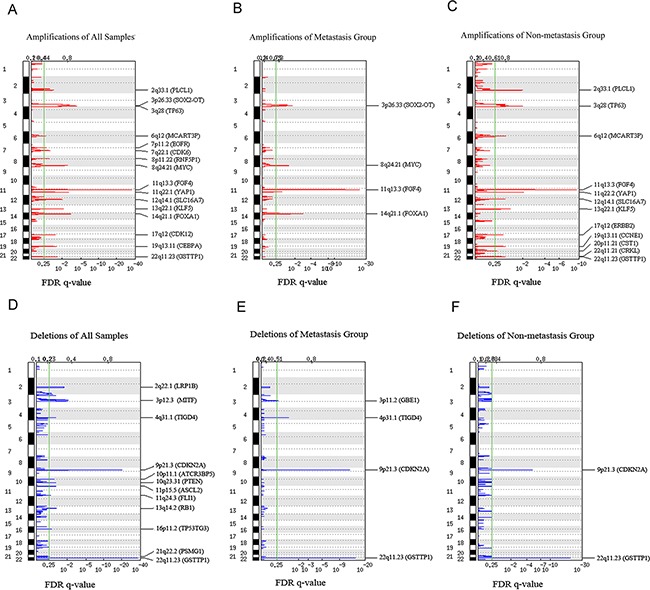
GISTIC analysis of copy number alterations in esophageal squamous cell carcinoma (ESCC) False discovery rate (FDR) q-values are plotted along the x axis with chromosomal position along the y axis. Regions with q values < 0.25 (green lines) were considered significantly altered. Known or putative gene targets within the peak regions and the regions are indicated for significant peaks. **A**. Amplifications of all superficial ESCCs. **B**. Amplifications of superficial ESCCs with metastasis. **C**. Amplifications of superficial ESCCs without metastasis. **D**. Deletions of all superficial ESCC. **E**. Deletions of superficial ESCCs with metastasis. **F**. Deletions of superficial ESCCs without metastasis.

We found 28 significantly recurrent focal CNAs, including 16 amplifications and 12 deletions in all 38 superficial ESCCs (Supplementary Table S1) (Figure 2). The most common CNAs were amplifications of 11q13.3 (*FGF4*), 3q28 (*TP63*), 14q21.1 (*FOXA1*), 8q24.21 (*MYC*), and deletions of 9p21.3 (*CDKN2A*), 22q11.23 (*GSTT1*), 3p13 (*MITF*) and 2q22.1 (*LRP1B*). In metastasis group, eight recurrent focal CNAs were found, including 4 amplifications and 4 deletions (Supplementary Table S2). The most common CNAs were amplifications of 11q13.3 (*FGF4*), 14q21.1 (*FOXA1*), 3q26.33 (*SOX2-OT*), 8q24.21 (*MYC*) and deletions of 22q11.23 (*GSTT1*), 9p21.3 (*CDKN2A*). In non-metastasis group, fourteen recurrent focal CNAs were found, including 12 amplifications and 2 deletions (Supplementary Table S3). The most common CNAs were amplifications of 11q13.3 (*FGF4*), 2q33.1 (*PLCL1*), 3q28 (*TP63*) and deletions of 22q11.23 (*GSTT1*) and 9p21.3 (*CDKN2A*). In conclusion, amplifications of 11q13.3 (*FGF4*), 8q24.21 (*MYC*) and deletions of 9p21.3 (*CDKN2A*) and 2q22.1 (*LRP1B*), which previously reported in ESCCs were also found in all 38 superficial ESCCs in our study. Furthermore, deletions of 3p13 (*MITF*) and 22q11.23 (*GSTT1*) were also found as CNAs targets of ESCC but were not previously reported. Amplification of 3q26.33 (*SOX2-OT*) was the most significantly recurrent CNA in metastasis group whereas amplification of 3q28 (*TP63*) was the most significantly recurrent CNA in non-metastasis group.

### Comparison of CNAs between metastasis and non-metastasis groups of 891 cancer genes

**Figure 3:**
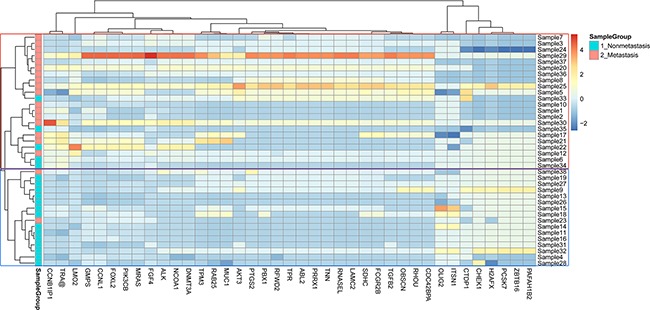
Heat map generated by unsupervised hierarchical clustering based on contributions of copy number alteration profiles of 39 gene signatures identified by Mann-Whitney U-test in 38 esophageal squamous cell carcinoma genomes The blue and red color columns represent non-metastasis and metastasis groups respectively.

Given Oncoscan microarray has increased probe density within 891 cancer genes, these 891 cancer genes were analyzed separately. Among these 891 genes, 39 genes had significantly different CNAs between metastasis and non-metastasis groups (Mann-Whitney U-test, *p* < 0.05). Hierarchical clustering was performed on the CNA profiles of these 39 genes and two groups were obtained, including metastasis and non-metastasis groups. In general, good separated performance of metastasis and non-metastasis was obtained with a small amount of misclassification (two metastasis misclassified and five non-metastasis misclassified) (Figure 3).

**Figure 4:**
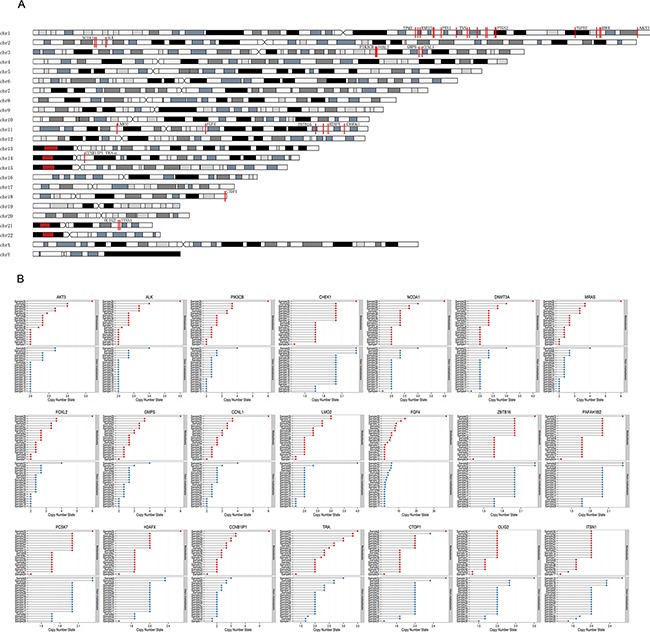
The genomic distribution and copy number of 39 genes identified by Mann-Whitney U-test in 38 esophageal squamous cell carcinoma genomes **A**. The red lines marked on chromosomes represented the position of the 39 genes on genome. **B**. The copy numbers of the part of the 39 genes in metastasis and non-metastasis groups were shown. The copy numbers of other part of the 39 genes were shown in Supplementary Figure S2.

The genomic distribution and copy numbers of these 39 genes was shown in Figure 4 and Supplementary Figure S1. The comparison of the 39 genes was shown in Table 1. Among the 39 genes, average copy number of *FGF4* in all cases (11q13.3, Mann-Whitney U-test, *p* = 0.045) were 5.14. Amplification of *FGF4* happened in 68% cases in metastasis group, and 58% in non-metastasis group. *FGF4* gene was located on chromosome 11q13.3, which were found to be amplified in ESCCs from previous studies. Average copy number of *MRAS* gene (3q22.3, Mann-Whitney U-test, *p* = 0.029) in all cases was 2.61, and 68% cases had amplification of *MRAS* gene in metastasis and 84% cases had amplifications in non-metastasis group. Average copy number of *ALK* (2p21, Mann-Whitney U-test, *p* = 0.024) in all cases was 2.25. Amplification of *ALK* gene happened in 26% of non-metastasis cases, and 63% of metastasis cases. Average copy number of *CHEK1* (11q24.2, Mann-Whitney U-test, *p* = 0.017) in all cases was 1.91. In non-metastasis group 3 cases (16%) had amplification of *CHEK1* and 3 cases (16%) had deletion of *CHEK1*, and in metastasis group only one case had amplification and 10 cases (53%) had deletion of *CHEK1*.

**Table 1:** The significantly different copy numbers of thirty nine genes in metastasis and non-metastasis groups

Gene name	Position	Average CN	P value	Non-metastasis			Metastasis		
				Average CN	Amplification	Deletion	Average CN	Amplification	Deletion
**TPM3**	Chr1:154117779-154165725	2.32	0.021	2.16	8 (0.42)	0 (0)	2.49	13 (0.68)	0 (0)
**MUC1**	Chr1:155148299-155172706	2.28	0.023	2.14	8 (0.42)	1 (0.05)	2.42	13 (0.68)	0 (0)
**RAB25**	Chr1:156020966-156050295	2.31	0.029	2.16	8 (0.42)	1 (0.05)	2.46	13 (0.68)	0 (0)
**SDHC**	Chr1:161274165-161344535	2.35	0.021	2.17	8 (0.42)	0 (0)	2.53	14 (0.74)	0 (0)
**FCGR2B**	Chr1:161622904-161658444	2.34	0.022	2.17	8 (0.42)	0 (0)	2.51	14 (0.74)	0 (0)
**PBX1**	Chr1:164518596-164831060	2.32	0.049	2.17	8 (0.42)	0 (0)	2.46	13 (0.68)	0 (0)
**PRRX1**	Chr1:170623312-170718541	2.30	0.023	2.14	7 (0.37)	0 (0)	2.46	13 (0.68)	0 (0)
**TNN**	Chr1:175026993-175127202	2.30	0.023	2.14	7 (0.37)	0 (0)	2.46	13 (0.68)	0 (0)
**RFWD2**	Chr1:175903966-176186370	2.28	0.025	2.12	7 (0.37)	1 (0.05)	2.45	13 (0.68)	0 (0)
**ABL2**	Chr1:179058461-179122224	2.30	0.023	2.14	7 (0.37)	0 (0)	2.46	13 (0.68)	0 (0)
**RNASEL**	Chr1:182532768-182568394	2.29	0.023	2.14	7 (0.37)	0 (0)	2.44	13 (0.68)	0 (0)
**LAMC2**	Chr1:183145173-183220406	2.29	0.023	2.14	7 (0.37)	0 (0)	2.44	13 (0.68)	0 (0)
**TPR**	Chr1:186270785-186354457	2.28	0.045	2.14	7 (0.37)	0 (0)	2.42	12 (0.63)	0 (0)
**PTGS2**	Chr1:186630943-186659559	2.30	0.034	2.14	7 (0.37)	0 (0)	2.46	12 (0.63)	0 (0)
**TGFB2**	Chr1:218508675-218627961	2.28	0.045	2.14	7 (0.37)	0 (0)	2.42	12 (0.63)	0 (0)
**CDC42BPA**	Chr1:227167565-227515826	2.30	0.043	2.16	6 (0.32)	0 (0)	2.44	12 (0.63)	0 (0)
**OBSCN**	Chr1:228385860-228558951	2.30	0.043	2.16	6 (0.32)	0 (0)	2.44	12 (0.63)	0 (0)
**RHOU**	Chr1:228770393-228892416	2.30	0.043	2.16	6 (0.32)	0 (0)	2.44	12 (0.63)	0 (0)
**AKT3**	Chr1:243641534-244016584	2.26	0.047	2.14	6 (0.32)	0 (0)	2.39	12 (0.63)	0 (0)
**NCOA1**	Chr2:24797345-25003570	2.23	0.036	2.10	5 (0.26)	1 (0.05)	2.35	11 (0.58)	0 (0)
**DNMT3A**	Chr2:25445829-25485184	2.23	0.036	2.10	5 (0.26)	1 (0.05)	2.35	11 (0.58)	0 (0)
**ALK**	Chr2:29405639-30154477	2.25	0.024	2.12	5 (0.26)	0 (0)	2.37	12 (0.63)	0 (0)
**MRAS**	Chr3:138056489-138134377	2.61	0.029	2.37	13 (0.68)	0 (0)	2.86	16 (0.84)	0 (0)
**PIK3CB**	Chr3:138361539-138436463	2.62	0.029	2.37	13 (0.68)	0 (0)	2.88	16 (0.84)	0 (0)
**FOXL2**	Chr3:138653065-138675982	2.62	0.043	2.39	13 (0.68)	0 (0)	2.86	16 (0.84)	0 (0)
**GMPS**	Chr3:155578324-155665520	2.74	0.042	2.51	15 (0.79)	0 (0)	2.96	18 (0.95)	0 (0)
**CCNL1**	Chr3:156855585-156888482	2.75	0.032	2.51	15 (0.79)	0 (0)	2.98	18 (0.95)	0 (0)
**LMO2**	Chr11:33870122-33901371	2.15	0.031	2.05	2 (0.11)	4 (0.21)	2.25	9 (0.47)	2 (0.11)
**FGF4**	Chr11:69577796-69600171	5.14	0.045	3.15	11 (0.58)	0 (0)	7.12	13 (0.68)	0 (0)
**ZBTB16**	Chr11:113920430-114131397	1.90	0.049	1.97	2 (0.11)	4 (0.21)	1.83	1 (0.05)	10 (0.53)
**PAFAH1B2**	Chr11:117004999-117051761	1.90	0.049	1.97	2 (0.11)	4 (0.21)	1.83	1 (0.05)	10 (0.53)
**PCSK7**	Chr11:117065787-117112811	1.90	0.049	1.97	2 (0.11)	4 (0.21)	1.83	1 (0.05)	10 (0.53)
**H2AFX**	Chr11:118954584-118976177	1.89	0.041	1.95	2 (0.11)	4 (0.21)	1.83	1 (0.05)	11 (0.58)
**CHEK1**	Chr11:125485030-125537042	1.91	0.017	2.00	3 (0.16)	3 (0.16)	1.83	1 (0.05)	10 (0.53)
**CCNB1IP1**	Chr14:20769528-20807533	2.36	0.039	2.14	6 (0.32)	2 (0.11)	2.58	12 (0.63)	1 (0.05)
**TRA@**	Chr14:22080057-23031075	2.29	0.040	2.13	6 (0.32)	3 (0.16)	2.45	12 (0.63)	1 (0.05)
**CTDP1**	Chr18:77429800-77524510	1.91	0.041	1.98	2 (0.11)	3 (0.16)	1.84	2 (0.11)	11 (0.58)
**OLIG2**	Chr21:34388215-34411503	1.94	0.036	2.04	4 (0.21)	3 (0.16)	1.84	0 (0)	7 (0.37)
**ITSN1**	Chr21:35004783-35220802	1.94	0.030	2.03	4 (0.21)	3 (0.16)	1.85	0 (0)	7 (0.37)

The most significantly enriched pathway/functional terms were “Proto-oncogene” (*p* = 7.59E-11), “Chromosomal rearrangement” (*p* = 8.64E-9), “Pathways in cancer” (*p* = 6.66E-04) and “Nucleotide-binding” (*p* = 0.015).

## DISCUSSION

The main purpose of this study is to identify CNAs that could differentiate superficial ESCC patients with high risk of metastasis. Although several whole genome copy number analyses on ESCC have been reported, there has been no systematic study on superficial ESCC. In this study, we used Affymetrix OncoScan™ (SNP microarray) to analyze genome wide CNAs of 38 superficial ESCCs and compared recurrent focal CNAs level, and the CNAs of 891 cancer genes between ESCCs with and without metastasis.

The Oncoscan SNP microarray is an ideal platform to perform analyses on FFPE-derived tumor materials with increased probe density within 891 cancer genes at 50-100 kb resolution. Among the 891 cancer genes, 39 cancer genes had significantly different CNAs between the metastasis and non-metastasis cases. These genes might represent candidate biomarkers for superficial ESCC patients with high risk of metastasis after surgery. Previous studies focus on using gene expression profiles to guide prognosis prediction and diagnosis [18, 19]. Here we suggested that DNA copy number may be used for the same purpose with the advantage of being more stable [20]. *FGF4*, *PIK3CB*, *MRAS*, *ALK*, *LMO2*, *AKT3* and *CHEK1* genes were included in the 39-gene signature. Copy number of *CHEK1* gene was significantly lower in metastasis than in non-metastasis groups (*p* = 0.017). Checkpoint kinase 1 (Chk1) which encoded by *CHEK1* gene trigger cell cycle arrest upon DNA damage [21] Although *CHEK1* was initially thought to be a tumor suppressor gene due to its role in checkpoint activation and cell cycle arrest, several studies documented a positive correlation between Chk1 expression and tumor grade and disease recurrence, suggesting that Chk1 may promote tumor growth [22–24]. So the role of *CHEK1* in ESCC should be explored further. In addition, *FGF4* gene located in 11q13.3 was also recurrently amplified in superficial ESCC.

In discovery and validation experiments, there were 88.5% (23/26) cases showed the identical copy number aberrations of *CCNL1* gene, and 76% (19/25) cases for *PIK3CB* gene. The difference of calculation of copy number alterations in these two experiments might lead to inconsistency of results.

The recurrent focal CNAs which determined by GISTIC2.0 were more likely to identify cancer-causing genes [14, 25]. Among all 38 superficial ESCC samples, recurrent focal amplifications of 11q13.3 (*FGF4* and *FGF19*), 8q24.21 (*MYC*), 7q22.1 (*CDK6*) and deletions of 9p21.3 (*CDKN2A*), 3p12.3 (*MITF*), 2q22.1 (*LRP1B*), 13q14.2 (*RB1*) which were previously reported in all stage of ESCC [8, 11, 12, 26–30] were also found in our study. This indicated that those CNAs might be the driver events in early stage of ESCC. In addition, amplification of 11q21.1 (*FOXA1*), which has not been previously reported as associated with ESCCs, was found in 20 cases (52.6%) in our study. Forkhead box protein A1 (*FOXA1*), the representative member of the Forkhead-box (FOX) proteins subfamily, is a DNA-binding transcription factor. Amplification of *FOXA1* gene has been found in lung cancer, esophageal adenocarcinoma, [31] estrogen receptor (ER)-positive breast cancer [32] and anaplastic thyroid cancer [33]. And the levels of FOXA1 protein have been correlated with the prognosis of breast cancer and gastric cancer [34, 35]. Deletions of 22q11.23 (*GSTT1*, *GSTTP1*, and *GSTT2*) occurred in 20 superficial ESCC samples (57.9%) in our study. Glutathione S-transferase (GST) theta 1 (GSTT1) which encoded by *GSTT1* gene is a member metabolizing dimeric phase II enzymes superfamily. These enzymes play a vital role in cellular defense system by catabolism of a broad range of xenobiotics and carcinogen [36, 37]. Many previous studies have found that *GSTT1* gene deletion polymorphisms increases susceptibility to lymphoma, breast cancer, colon cancer and lung cancer [38–40]. So the results of our study suggested that the role of *GSTT1* deletion in the etiology of ESCC should be explored further.

Amplifications of 3p26.33 (*SOX2-OT*), 8q24.21 (*MYC*), 14q21.1 (*FOXA1*) and deletion of 3p12.1 (*GBE1*) were found as the recurrent CNAs in metastasis group only, which indicated that those CNAs might be associated with metastasis in superficial ESCC. Deletion of 3p12.1 (*GBE1*) has been associated with prognosis in cervical cancer [41], but not in ESCC previously. Sex determining region Y-box 2 (SOX2), a key transcription factor involved in self-renewal and pluripotency of embryonic stem cells, plays an important role in tumor cell metastasis and apoptosis [42]. *SOX2* gene embedded in the introns of *SOX2* overlapping transcript (*SOX2OT*) gene, which encodes a long non-coding RNA (lncRNA) [43]. Amplifications of *SOX2* have been associated with metastasis or poor prognosis in previous studies of ESCC [9, 44–46]. In our study, amplification of *SOX2OT* gene on 3p26.33 was found as recurrent CNA in metastasis group. *SOX2OT* gene encodes the lncRNA which have been demonstrated that involved in regulation of *SOX2* expression and/or other related processes. And expression of *SOX2* and *SOX2OT* were concordant in ESCC and breast cancer [47, 48]. The role of *SOX2OT* gene and expression in ESCC metastasis should be explored further.

In conclusion, our study constructed a 39-gene signature associated with metastasis from superficial ESCCs. The comparison of recurrent CNAs between superficial ESCCs with and without metastasis also revealed a subset of metastasis specific events. A larger set of independent samples are warranted to validate and refine this 39-gene signature from our study.

## MATERIALS AND METHODS

### Patients and genomic DNA extraction

Thirty eight superficial ESCC patients, at stage T1N0M0, were collected from Cancer Hospital, Chinese Academy of Medical Sciences between 2004 and 2010. The most recent follow-up visit was dated in June 2015 and the follow was conducted via telephone interview or clinical data consultation. Among the 38 cases, 19 metastasis cases have lymph node metastasis or distant metastasis within five years from radical surgery, and the 19 metastasis-free cases have no detectably metastasis within five years after radical surgery. All the patients underwent radical resection without radiotherapy or chemotherapy prior to surgery. The clinicopathological characters of 38 patients were summarized in Table 2. The Institute Review Board of the Cancer Hospital, CAMS, agreed to waive the need for consent for this study and approved the study protocol.

**Table 2:** Clinicopathological features of 38 superficial ESCC cases

Features		Non-metastasis	Metastasis
**Gender**	Female	7	7
	Male	12	12
**Age**	>=57	10	13
	<57	9	6
**Family history**	Yes	4	4
	No	15	15
**Smoking**	Yes	14	9
	No	5	10
**Drinking**	Yes	12	5
	No	7	14
**Differentiation**	Well	6	5
	Moderately	12	12
	Poorly	1	2
**Location**	Upper thoracic	1	3
	Middle thoracic	7	7
	Lower thoracic	11	9
**Depth**	mucosa	1	0
	Submucosa	18	19
**Lymphovascular invasion**	Yes	1	2
	No	18	17

Genomic DNA were extracted from formalin-fixed and paraffin-embedded (FFPE) tissues of 38 superficial ESCCs using QIAamp DNA Mini kit (Qiagen, Hilgen, Germany) according to the manufacturer's instruction. H&E sections of all cases were reviewed to manually identify areas with minimum of 85% malignant cells for microdissection.

### Single nucleotide polymorphism (SNP) array

Molecular Inversion Probe (MIP) based Oncoscan Array was used to detect CNAs, loss of heterozygosity, and somatic point mutations. The experiments were performed according to the user guide of Affymetrix OncoScan™ FFPE Assay Kit (Affymetrix, CA). Briefly, 75 ng FFPE DNA were hybridized to MIP probes and allowed to anneal 58°C overnight (16-18h) after denaturation at 95°C for 5 min. Then each sample was split into two tubes and gap fill reaction was performed by adding dATP (A) and dTTP (T) (A/T) in one tube and dGTP (G) and dCTP (C) (G/C) to another. After removing the un-circularized MIP probes through exonuclease treatment, the cleavage enzyme was added to linearize the gap-filled circular MIP Probes. Then the circular MIP probes were amplified by first and second round PCR. The enriched product was digested by HaeIII enzyme and the 44bp fragments were hybridized to the OncoScan™ Array for 16-18h. The hybridized arrays were washed, stained using the GeneChip® Fluidics Station 450 and scanned through GeneChip® Scanner 3000 7G (Affymetrix, CA).

### Real time PCR (qPCR)

Real Time PCR (qPCR) was used to validate CNAs of *CCNL1* and *PIK3CB* genes with the internal control *HBB* gene using SYBR-Green II fluorescence and Mx3005P System (Agilent Technologies, CA, USA)) (Supplementary Table S4). The results were analysed using the MxPro QPCR software. Comparative CT method was used to calculate the copy numbers of target genes. Cases with 2^−ΔΔCT^ > 1 were considered as amplification of genes, and cases with 2^−ΔΔCT^ < 1 were considered as deletion of genes [49].

### Data and statistical analysis

The intensity (CEL) files generated by the scanner were imported into Oncoscan Console Software (Biodiscovery, Inc., CA USA) and analyzed by the Affymetrix TuScan algorithm (a modified ASCAT algorithm) to create segmentation to differentiate between adjacent clusters of probes and determines the CNAs. Amplification was defined as copy number which was calculated using Affymetrix TuScan algorithm > 2, and copy number < 2 was considered to be deletion. In addition, the copy number information of 891 cancer genes were obtained as Gene Report text files from the Console software.

The recurrent CNAs were determined using GISTIC2.0 (Genomic Identification of Significant Targets in Cancer) with a Q-value cutoff < 0.25. And the significant recurrent focal CNAs has a 90% likelihood of containing the targeted genes.

The Mann-Whitney U-test was used to detect the significantly different CNAs of 891 cancer genes between metastasis group and non-metastasis group. And then, hierarchical clustering was performed on significantly differential CNAs of cancer genes. Pheatmap function in R pheatmap package was used to draw heat maps to visualize the clustering results.

Genome instability index (GII) was used to evaluate the levels of DNA copy number changes in all 38 samples. To take the variation of chromosome size into consideration, weighted GII was used. First, percentage of aberrant SNPs for each chromosome was calculated separately to obtain GII of each chromosome. For individual chromosome, numbers of SNPs with aberrant copy number were divided by numbers of all SNPs in each chromosome to get the GII of each chromosome. Then the mean percentage aberration of all 22 autosomes in each sample was calculated to generate the weighted GII [50, 51]. Paired t-test was used to compare the difference between metastasis and non-metastasis groups (*p* < 0.05). All the statistical analyses were performed on R (version 3.2.1). Significance were deemed as < 0.05 for all statistical analyses.

Functional enrichment analyses was performed on the 39 genes using DAVID (Database for Annotation, Visualization and Integrated Discovery) tool. These terms achieved significant enrichment p values after adjusted with the Benjamini method.

## SUPPLEMENTARY FIGURES AND TABLES








