# Supplementary material for: Genome wide copy number analyses of superficial esophageal squamous cell carcinoma with and without metastasis
Source: Oncotarget. 2016 Dec 10;8(3):5069–80. doi: 10.18632/oncotarget.13847 (PMC5354893; doi:10.18632/oncotarget.13847)
Supplement: Supplementary file 1 [file oncotarget-08-5069-s001.pdf]

Genome wide copy number analyses of superficial esophageal squamous cell carcinoma with and without metastasis

SUPPLEMENTARY FIGURES AND TABLES

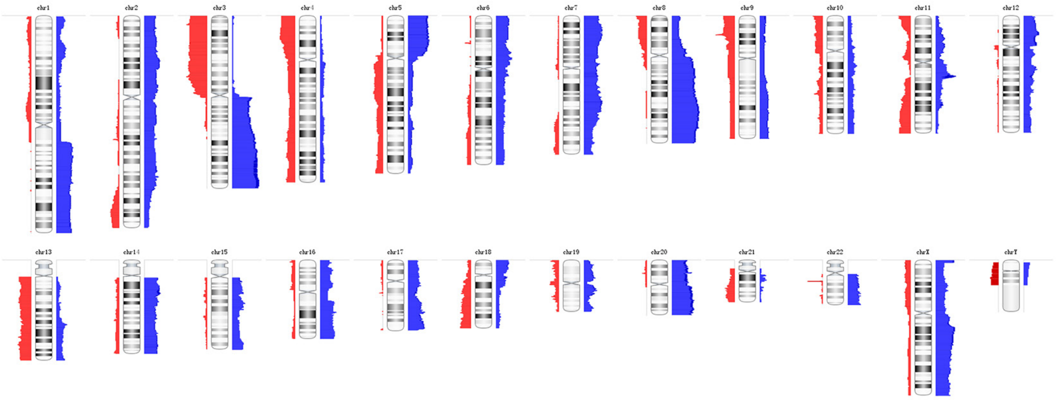

Supplementary Figure S1: Genome wide copy number alteration profiles of esophegal squamous cell carcinoma.

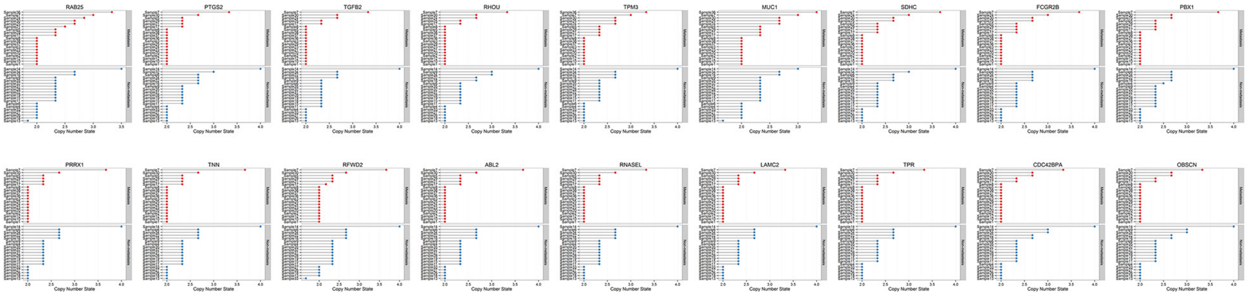

Supplementary Figure S2: Copy number of 38 significantly different cancer genes.

Supplementary Table S1: Amplification and deletion in 38 superficial ESCC.

See Supplementary File 1

Supplementary Table S2: Amplification and deletion in superficial ESCC metastasis group

Aberration	Cytoband	q value	Residual q value	Wide peak boundaries	Genes in wide peak
amplification	11q13.3	6.07E-25	6.07E-25	chr11:69505646-70229170	hsa-mir-548k FGF3 FGF4 PPFIA1 FADD FGF19 ANO1 MIR548K
amplification	14q21.1	0.001262	0.001262	chr14:37723237-38345848	FOXA1 MIPOL1
amplification	3q26.33	0.019159	0.019159	chr3:181522021-181879655	SOX2-OT
amplification	8q24.21	0.0385	0.0385	chr8:128756748-128785045	MYC
deletion	22q11.23	1.41E-15	1.41E-15	chr22:24314259-24408077	GSTT1 GSTT2 GSTTP1 LOC391322 GSTTP2
deletion	9p21.3	2.56E-13	2.56E-13	chr9:21864099-21995085	CDKN2A C9orf53
deletion	4q31.3	0.056146	0.056146	chr4:153421335-153701129	DKFZP434I0714 TIGD4 TMEM154 MIR4453
deletion	3p12.1	0.22579	0.22579	chr3:79816313-93597666	EPHA3 GBE1 HTR1F POU1F1 CGGBP1 CHMP2B ZNF654 CADM2 C3orf38 VGLL3 LOC440970 MIR4795

Supplementary Table S3: Amplification and deletion in superficial ESCC non-metastasis group.

See Supplementary File 2

Supplementary Table S4: Copy numbers of CCNL1 gene and PIK3CB gene of real time PCR.

See Supplementary File 3
