# Supplementary material for: Genome wide copy number analyses of superficial esophageal squamous cell carcinoma with and without metastasis
Source: Oncotarget. 2016 Dec 10;8(3):5069–80. doi: 10.18632/oncotarget.13847 (PMC5354893; doi:10.18632/oncotarget.13847)
Supplement: Supplementary file 2 [file oncotarget-08-5069-s002.docx]

**Supplementary Table 1. Amplification and deletion in 38 superficial ESCC**

| Aberration | Cytoband | q value | Residual q value | Wide peak boundaries | Genes in wide peak |
| --- | --- | --- | --- | --- | --- |
| amplification | 11q13.3 | 3.25E-36 | 5.36E-36 | chr11:69511946-69603217 | FGF4 FGF19 |
| amplification | 14q21.1 | 0.000757 | 0.000757 | chr14:37723237-38345848 | FOXA1 MIPOL1 |
| amplification | 8q24.21 | 0.001995 | 0.001995 | chr8:128561432-128709993 | MYC |
| amplification | 3q28 | 7.00E-05 | 0.005425 | chr3:188893134-189440331 | TP63 TPRG1 |
| amplification | 13q22.1 | 0.005986 | 0.005986 | chr13:73700540-74120294 | KLF5 |
| amplification | 12q14.1 | 0.008569 | 0.008569 | chr12:60726426-60882826 | SLC16A7 |
| amplification | 3q26.33 | 0.00015 | 0.02291 | chr3:181522021-181879655 | SOX2-OT |
| amplification | 22q11.23 | 0.026813 | 0.026813 | chr22:24338652-24390317 | GSTT1 GSTTP1 LOC391322 GSTTP2 |
| amplification | 19q13.11 | 0.034887 | 0.034887 | chr19:31463349-34507252 | CEBPA CEBPG LRP3 PEPD PDCD5 SLC7A9 ZNF507 GPATCH1 SLC7A10 TSHZ3 CHST8 KCTD15 LOC80054 ANKRD27 CEP89 RHPN2 C19orf40 TDRD12 DKFZp566F0947 WDR88 DPY19L3 RGS9BP NUDT19 LOC400684 THEG5 |
| amplification | 17q12 | 0.043271 | 0.043271 | chr17:37656953-37767452 | NEUROD2 |
| amplification | 2q33.1 | 0.056973 | 0.056973 | chr2:198022928-200658050 | HSPD1 HSPE1 PLCL1 SATB2 SF3B1 MOB4 BOLL COQ10B ANKRD44 MARS2 RFTN2 FLJ32063 FONG HSPE1-MOB4 |
| amplification | 7q22.1 | 0.10752 | 0.10752 | chr7:87497371-108817211 | hsa-mir-548o hsa-mir-4285 hsa-mir-106b hsa-mir-591 hsa-mir-489 hsa-mir-1285-1 ACHE ASNS AZGP1 CALCR KRIT1 CDK6 AP1S1 COL1A2 CUX1 CYP3A7 CYP3A4 CYP3A5 CYP51A1 DLD DLX5 DLX6 DYNC1I1 SLC26A3 EPHB4 EPO GNB2 GNG11 GNGT1 GPR22 AGFG2 LAMB1 LRCH4 MCM7 DNAJB9 NPTX2 NRCAM OCM2 ORC5 SERPINE1 PCOLCE PDK4 SLC26A4 PEX1 CDK14 PIK3CG PMS2P1 POLR2J PON1 PON2 PON3 PRKAR2B RELN PSMC2 SRI SRPK2 SYPL1 TAC1 TAF6 TFR2 TRIP6 VGF ZAN ZNF3 ZKSCAN1 ZSCAN21 MTERF SHFM1 TFPI2 TRRAP FZD1 BUD31 SGCE PLOD3 CLDN12 AP4M1 PMPCB ATP5J2 MUC12 ARPC1B NAMPT AKAP9 RASA4 SLC25A13 LRRC17 POP7 BET1 COG5 ZNHIT1 ARPC1A SH2B2 STAG3 CPSF4 DBF4 COPS6 DUS4L PDAP1 |
| amplification | 8p11.22 | 0.11878 | 0.11878 | chr8:38406808-38474483 | RNF5P1 |
| amplification | 11q22.1 | 0.005986 | 0.13199 | chr11:101003113-102789004 | BIRC2 BIRC3 MMP1 MMP3 MMP7 MMP8 MMP10 MMP12 TRPC6 MMP20 YAP1 KIAA1377 MMP27 C11orf70 TMEM123 ANGPTL5 LOC100288077 MIR3920 |
| amplification | 7p11.2 | 0.17665 | 0.17665 | chr7:54337149-55333390 | EGFR SEC61G VSTM2A LOC285878 |
| amplification | 6q12 | 0.20123 | 0.20123 | chr6:67245185-67369247 | MCART3P |
| deletion | 22q11.23 | 1.92E-37 | 1.92E-37 | chr22:24314259-24388237 | GSTT1 GSTT2 GSTTP1 LOC391322 |
| deletion | 9p21.3 | 5.21E-21 | 5.21E-21 | chr9:21864099-21995085 | CDKN2A C9orf53 |
| deletion | 3p12.3 | 0.007049 | 0.007049 | chr3:69413122-93597666 | hsa-mir-4273 hsa-mir-1324 hsa-mir-1284 EPHA3 GBE1 GPR27 HTR1F MITF CNTN3 POU1F1 ROBO1 ROBO2 CGGBP1 PDZRN3 RYBP CHMP2B FOXP1 EBLN2 SHQ1 ZNF654 PROK2 PPP4R2 LOC201617 CADM2 C3orf38 EIF4E3 VGLL3 LOC401074 LOC440970 FLJ20518 FAM86DP GXYLT2 ZNF717 FRG2C MIR1284 MIR1324 MIR4273 MIR4795 MIR4444-1 |
| deletion | 2q22.1 | 0.020529 | 0.020819 | chr2:139645075-143647318 | LRP1B LOC647012 |
| deletion | 13q14.2 | 0.088862 | 0.088862 | chr13:42543964-57748969 | hsa-mir-1297 hsa-mir-759 hsa-mir-15a ATP7B RCBTB2 CPB2 ESD MLNR GTF2F2 GUCY1B2 HTR2A KPNA3 LCP1 NEK3 PCDH8 RB1 TPT1 TNFSF11 SUCLA2 DLEU2 TSC22D1 ITM2B UTP14C LPAR6 TRIM13 DLEU1 OLFM4 SUGT1 LECT1 AKAP11 FNDC3A ZC3H13 LRCH1 INTS6 CKAP2 NUFIP1 MED4 DNAJC15 VPS36 PHF11 ENOX1 RCBTB1 NUDT15 KIAA1704 THSD1 CYSLTR2 SPRYD7 RNASEH2B DHRS12 KIAA0226L CDADC1 CAB39L CCDC70 COG3 SETDB2 EBPL EPSTI1 ARL11 WDFY2 LINC00284 PRR20A FAM216B LACC1 LINC00330 HNRNPA1L2 ST13P4 DGKH CCDC122 FAM194B SPERT DLEU7 FAM124A TPTE2P3 CTAGE10P SLC25A30 SIAH3 KCNRG LINC00282 NEK5 THSD1P1 KCTD4 SERP2 MIR15A MIR16-1 ALG11 TSC22D1-AS1 SERPINE3 SNORA31 PRR20B PRR20C PRR20D PRR20E TPT1-AS1 MIR1297 MIR759 MIR3613 LOC100509894 MIR4703 |
| deletion | 4q31.3 | 0.091073 | 0.091073 | chr4:153400875-153701129 | hsa-mir-3140 DKFZP434I0714 TIGD4 TMEM154 MIR3140 MIR4453 |
| deletion | 10q23.31 | 0.094449 | 0.094449 | chr10:89621062-90038032 | PTEN |
| deletion | 11p15.5 | 0.094449 | 0.094449 | chr11:1696017-3024383 | hsa-mir-483 hsa-mir-675 hsa-mir-4298 ASCL2 CD81 CDKN1C CTSD IGF2 INS KCNQ1 LSP1 NAP1L4 SLC22A18 SLC22A18AS MRPL23 TH TNNI2 TNNT3 PHLDA2 TSPAN32 TSSC4 KCNQ1OT1 C11orf21 TRPM5 IGF2-AS1 KCNQ1DN SYT8 H19 IFITM10 KRTAP5-6 MIR483 SNORA54 INS-IGF2 MIR675 FAM99B MRPL23-AS1 MIR4298 MIR4686 |
| deletion | 10p11.1 | 0.1182 | 0.12239 | chr10:38674873-42832211 | LOC399744 ACTR3BP5 |
| deletion | 16p11.2 | 0.17565 | 0.18865 | chr16:31928621-34197194 | hsa-mir-1826 TP53TG3 SLC6A10P LOC390705 HERC2P4 LINC00273 TP53TG3C LOC729264 TP53TG3B |
| deletion | 11q24.3 | 0.20479 | 0.18953 | chr11:126864308-128712780 | ETS1 FLI1 KCNJ1 KIRREL3-AS3 LOC100507392 |
| deletion | 21q22.2 | 0.23575 | 0.23575 | chr21:40509654-40826304 | HMGN1 WRB PSMG1 BRWD1 LCA5L BRWD1-IT2 |
