# Supplementary material for: Genome wide copy number analyses of superficial esophageal squamous cell carcinoma with and without metastasis
Source: Oncotarget. 2016 Dec 10;8(3):5069–80. doi: 10.18632/oncotarget.13847 (PMC5354893; doi:10.18632/oncotarget.13847)
Supplement: Supplementary file 3 [file oncotarget-08-5069-s003.docx]

**Supplementary Table 3. Amplification and Deletion in superficial ESCC non-metastasis group**

| aberration | cytoband | q value | residual q value | wide peak boundaries | genes in wide peak |
| --- | --- | --- | --- | --- | --- |
| amplification | 11q13.3 | 4.05E-10 | 4.05E-10 | chr11:69546730-69571941 | FGF4 |
| amplification | 2q33.1 | 0.009902 | 0.009902 | chr2:198126798-200288717 | HSPD1 HSPE1 PLCL1 SATB2 SF3B1 MOB4 BOLL COQ10B ANKRD44 MARS2 RFTN2 HSPE1-MOB4 |
| amplification | 3q28 | 0.009902 | 0.009902 | chr3:188893134-189683064 | hsa-mir-944 TP63 LEPREL1 TPRG1 MIR944 |
| amplification | 13q22.1 | 0.054104 | 0.054104 | chr13:73662802-74120294 | KLF5 |
| amplification | 6q12 | 0.091423 | 0.091423 | chr6:67060397-67369247 | MCART3P |
| amplification | 12q14.1 | 0.095668 | 0.095668 | chr12:60726426-61079477 | SLC16A7 |
| amplification | 19q13.11 | 0.095668 | 0.095668 | chr19:30308386-34507252 | CCNE1 CEBPA CEBPG LRP3 PEPD URI1 PDCD5 ZNF536 SLC7A9 ZNF507 GPATCH1 SLC7A10 TSHZ3 CHST8 KCTD15 LOC80054 ANKRD27 CEP89 RHPN2 C19orf40 TDRD12 DKFZp566F0947 WDR88 DPY19L3 RGS9BP NUDT19 LOC400684 THEG5 |
| amplification | 17q12 | 0.11719 | 0.11719 | chr17:37656953-37887732 | ERBB2 NEUROD2 PNMT TCAP STARD3 CDK12 PPP1R1B MIEN1 PGAP3 MIR4728 |
| amplification | 11q22.2 | 0.00824 | 0.1388 | chr11:101484719-102789004 | BIRC2 BIRC3 MMP1 MMP3 MMP7 MMP8 MMP10 MMP12 MMP20 YAP1 KIAA1377 MMP27 C11orf70 TMEM123 ANGPTL5 LOC100288077 |
| amplification | 22q11.21 | 0.13485 | 0.14362 | chr22:21068879-21273195 | CRKL SERPIND1 PI4KA SNAP29 |
| amplification | 20p11.21 | 0.21256 | 0.21256 | chr20:21342379-24160279 | CST1 CST2 CST3 CST4 CST5 FOXA2 NKX2-2 PAX1 SSTR4 THBD CST8 XRN2 CD93 NXT1 NAPB GZF1 GGTLC1 CSTL1 CST9L CST9 LINC00261 CST11 CSTT LOC200261 LOC284788 NKX2-4 LOC100270679 |
| amplification | 22q11.23 | 0.095668 | 0.30204 | chr22:24338652-24390317 | GSTT1 GSTTP1 LOC391322 GSTTP2 |
| deletion | 22q11.23 | 4.80E-21 | 4.80E-21 | chr22:24314259-24388237 | GSTT1 GSTT2 GSTTP1 LOC391322 |
| deletion | 9p21.3 | 9.01E-06 | 9.01E-06 | chr9:21864099-22471831 | CDKN2A CDKN2B C9orf53 DMRTA1 CDKN2B-AS1 |
