# Supplementary material for: Genome wide copy number analyses of superficial esophageal squamous cell carcinoma with and without metastasis
Source: Oncotarget. 2016 Dec 10;8(3):5069–80. doi: 10.18632/oncotarget.13847 (PMC5354893; doi:10.18632/oncotarget.13847)
Supplement: Supplementary file 4 [file oncotarget-08-5069-s004.docx]

**Supplementary Table 4. Copy numbers of CCNL1 gene and PIK3CB gene of Real Time PCR**

| Sample | Copy number of CCNL1(Oncoscan) | HBB_Ct1 | CCNL1_Ct | CCNL1_ΔCt | CCNL1_ΔΔCt | Copy number of PIK3CB(Oncoscan) | HBB_Ct2 | PIK3CB_Ct | PIK3CB_ΔCt | PIK3CB1_ΔΔCt |
| --- | --- | --- | --- | --- | --- | --- | --- | --- | --- | --- |
| Sample1 | 3 | 34.09 | 26.43 | -7.66 | -8.7 | 2 | 34.09 | 29.41 | -4.68 | -5.18 |
| Sample4 | 2.67 | 29.65 | 24.99 | -4.66 | -5.5 | 2.33 | 29.65 | 26.45 | -3.2 | -3.34 |
| Sample5 | 3.33 | 29.48 | 24.45 | -5.03 | -5.87 | 3.33 | 29.48 | 25.72 | -3.76 | -3.9 |
| Sample7 | 3 | 30.31 | 25.21 | -5.1 | -5.94 | 2.33 | 30.31 | 27.03 | -3.28 | -3.42 |
| Sample8 | 2 | 35.05 | 28.56 | -6.49 | -7.33 | 2 | 35.05 | 31.98 | -3.07 | -3.21 |
| Sample10 | 2.33 | 31.16 | 26.16 | -5 | -6.04 | 2.33 | 31.16 | 27.48 | -3.68 | -4.18 |
| Sample11 | 2.67 | 39.9 | 28.83 | -11.07 | -11.91 | 2.33 | 39.9 | 34.29 | -5.61 | -5.75 |
| Sample12 | 2.33 | 34.47 | 29.69 | -4.78 | -5.62 | 2.67 | 34.47 | 32.13 | -2.34 | -2.48 |
| Sample13 | 2 | 34.01 | 26.55 | -7.46 | -8.3 | 2 | 34.01 | 31.37 | -2.64 | -2.78 |
| Sample14 | 2.67 | 28.93 | 25.02 | -3.91 | -4.75 | 2.33 | 28.93 | 25.8 | -3.13 | -3.27 |
| Sample16 | 2.67 | 29.56 | 25.19 | -4.37 | -5.41 | 2.33 | 29.56 | 25.42 | -4.14 | -4.64 |
| Sample17 | 2.67 | 33.08 | 25.58 | -7.5 | -8.54 | 2.67 | 33.08 | 28.05 | -5.03 | -5.53 |
| Sample18 | 2.67 | 29.07 | 25.26 | -3.81 | -4.34 | 2.67 | 29.07 | 26.12 | -2.95 | -3.48 |
| Sample20 | 3.33 | 30.75 | 24.97 | -5.78 | -6.82 | 3.33 | 30.75 | 26.12 | -4.63 | -5.13 |
| Sample21 | 3.33 | 31.86 | 25.87 | -5.99 | -7.03 | 3.66 | 31.86 | 27.04 | -4.82 | -5.32 |
| Sample22 | 4 | 32.28 | 27.1 | -5.18 | -5.29 | 4 | 0 | 39.18 | 39.18 | 38.68 |
| Sample23 | 2.67 | 38.84 | 29.51 | -9.33 | -9.86 | 2.67 | 38.84 | 0 | -38.84 | -39.34 |
| Sample24 | 2.33 | 29.52 | 25.86 | -3.66 | -4.19 | 2.33 | 29.52 | 27.29 | -2.23 | -2.73 |
| Sample26 | 2 | 25.51 | 22.95 | -2.56 | -3.6 | 2 | 25.51 | 23.76 | -1.75 | -2.25 |
| Sample27 | 2.33 | 31.11 | 25.83 | -5.28 | -6.32 | 2.33 | 31.11 | 26.37 | -4.74 | -5.24 |
| Sample30 | 3.67 | 24.36 | 23.49 | -0.87 | -1.4 | 3.67 | 24.47 | 23.57 | -0.9 | -1.4 |
| Sample33 | 2.33 | 30.81 | 28.2 | -2.61 | -3.14 | 2.67 | 30.81 | 28.37 | -2.44 | -2.94 |
| Sample34 | 2.33 | 29.43 | 26.52 | -2.91 | -3.95 | 2.33 | 29.43 | 25.9 | -3.53 | -4.03 |
| Sample35 | 2 | 28.64 | 25.21 | -3.43 | -3.96 | 2 | 28.64 | 25.45 | -3.19 | -3.69 |
| Sample37 | 2.67 | 30.86 | 26.37 | -4.49 | -5.02 | 2.67 | 30.86 | 27.29 | -3.57 | -4.07 |
| Sample38 | 2.33 | 26.46 | 24.03 | -2.43 | -2.96 | 2.33 | 26.46 | 24.42 | -2.04 | -2.54 |
